# Associations between physical activity, physical fitness, and body composition in adults living in Germany: a cross-sectional replication study

**DOI:** 10.1038/s41598-026-61611-6

**Published:** 2026-07-09

**Authors:** Raphael Schilling, Janis Fiedler, Janina Krell-Roesch, Alexander Woll, Steffen C. E. Schmidt

**Affiliations:** Institute of Sports and Sports Science, Karlsruhe Institute of Technology, Karlsruhe, Germany

**Keywords:** Muscle strength, Phase angle, Bioelectrical impedance analysis, Aging, Health care, Medical research, Physiology, Risk factors

## Abstract

Previous work showed that physical fitness (PF) is more strongly related to body composition (BC) than self-reported physical activity (PA) in adults. In this study, we provide post-COVID BC statistics and evaluate the reproducibility of previously observed associations between PA, PF, and BC. We analyzed cross-sectional data from 320 adults aged 34–82 years collected in 2025 and compared them with data from 2021. PA was assessed using a validated questionnaire. PF was measured through a standardized performance test battery and BC was obtained via bioelectrical impedance analysis. Associations between PA, PF, and BC were analyzed using sex-specific linear regression models. No significant differences in BC were found between 2021 and 2025. Participants had higher PA and muscular strength but lower coordination in 2025 compared to 2021. PF showed stronger associations with BC than PA. Muscular strength remained the most important predictor of BC and showed the strongest association with phase angle (males: β = 0.40, *p* < .001; females: β = 0.31, *p* = .002). The consistency of these associations across two independent samples from 2021 to 2025 indicates a robust pattern under different societal conditions and highlights the importance of PF for supporting healthy aging with regard to BC.

## Introduction

Body composition (BC) is closely linked to health during adulthood^1,2^. Physical activity (PA) and physical fitness (PF) are associated with BC^3,4^, but evidence on the interrelationship between PA, PF, and BC remains limited as only a few studies have examined all three constructs simultaneously.

In a previous cross-sectional analysis conducted during a low-incidence phase of the COVID-19 pandemic, PF showed stronger associations with BC than PA in adults living in Germany^5^. Since pandemic-related restrictions are known to have impacted PA behavior and lifestyle patterns^6^, it remains unclear whether these associations also persist under post-pandemic conditions.

Therefore, the present short report aimed to replicate the previously observed associations between PA, PF, and BC. To this end, we used data collected in 2025 from an adult sample drawn from the same rural German community. Based on previous findings, we expected PF to remain more strongly associated with BC than PA.

## Methods

### Design and sample

This study was conducted using a randomly selected sample from the residents’ registration offices in Bad Schönborn, Germany, as part of an ongoing community-based longitudinal study established in 1992. Written informed consent was obtained from all participants, and the study was approved by the ethics committee of the Karlsruhe Institute of Technology. All methods were performed in accordance with the Declaration of Helsinki.

In 2025, 1.328 participants were initially invited. Of these, 424 finally participated and completed in-person assessments. Data on BC were available for 415 individuals. Of these, 41 participants had incomplete PF data (e.g., medical non-clearance or inability to complete motor testing). One participant was excluded because of physiologically implausible values (fat mass index (FMI) = 1.1 kg/m^2^) and another because of extreme values (> 3 standard deviations) relative to the sample distribution (fat-free mass index (FFMI) = 32.2 kg/m^2^). Of the remaining 372 participants, 52 were excluded due to incomplete PA data. The final sample thus comprised 320 adults.

### Physical activity

PA was assessed using a computerized questionnaire that demonstrated high reliability (Cronbach’s α = 0.94) as well as factorial validity and measurement invariance^7^. The questionnaire distinguished between sport-related and habitual activity (e.g., active transportation, gardening). Weekly minutes of sport activity were calculated from frequency and duration across different sports, while habitual activity was estimated from reported walking, cycling, and other physically demanding routines. Detailed scoring procedures have been described elsewhere^5^.

### Physical fitness

PF was measured by a standardized test battery^8^ comprising 15 motor performance tasks administered during a single session. Cardiorespiratory fitness (CRF) was estimated from a 2-km walking test; muscular strength by handgrip dynamometry (Takei Analog Hand Grip Dynamometer TKK-5001, 0–199 kg of force, Takei, Japan) and jump-and-reach; coordination by seven standardized motor tasks; and flexibility by sit-and-reach, side-bending, and three muscle extensibility tests. Performance values were converted into Z-scores to allow comparability across tests with different measurement units. Standardization was based on reference values from male participants aged 33 to 36 years assessed in 1992, as previously described^5,9^.

### Body composition

BC was assessed via bioelectrical impedance analysis (BIA) (seca mBCA 515, seca GmbH & Co. KG, Hamburg, Germany) according to international ESPEN standards^10^. Participants were invited to attend the assessment in a normal nutritional state. Using validated manufacturer algorithms^11,12^, BIA-derived phase angle (PhA), FMI (fat mass (kg)/height² (m)), and FFMI (fat-free mass (kg)/height² (m)) were calculated.

### Statistical analysis

Descriptive statistics were calculated, and differences between data collected in 2021 and 2025 were evaluated using 95% confidence intervals^13^. Associations of PA and PF with FMI, FFMI, and PhA were examined using multiple linear regression models. To allow for sex-specific interpretations, analyses were conducted separately for males and females. Crude models were first estimated, followed by models adjusted for age. Statistical significance was set at *p* < .05. All analyses were conducted using IBM SPSS Statistics 30 (IBM Corp., Armonk, NY, USA).

## Results

The 2025 sample comprised 320 adults (170 females, 150 males). Descriptive statistics of the 2021 and 2025 samples are presented in Table 1.

### Differences between waves 2021 and 2025

No significant differences were observed for FMI, FFMI, or PhA in 2025 compared with 2021. Both males and females reported higher sport and habitual activity in 2025, although only habitual activity reached statistical significance. Participants scored significantly higher in strength and lower in coordination tasks in 2025 than 2021.

**Table 1:** Descriptive statistics for the 2025 and 2021 samples.

	Year 2025		Year 2021	
	Males (*n* = 150) Mean ± SD [95% CI]	Females (*n* = 170) Mean ± SD [95% CI]	Males (*n* = 146) Mean ± SD [95% CI]	Females (*n* = 183) Mean ± SD [95% CI]
Anthropometrics & body composition				
Age (years)	57.1 ± 11.7 [55.2–59.0]	55.4 ± 11.2 [53.8–57.0]	57.7 ± 10.5 [56.0-59.4]	55.3 ± 10.6 [53.8–56.9]
Height (cm)	178.4 ± 7.2 [177.2-179.6]	164.4 ± 5.7 [163.5-165.3]	178.0 ± 7.1 [176.8-179.2]	164.5 ± 5.9 [163.6-165.4]
Weight (kg)	83.8 ± 12.1 [81.9–85.8]	66.1 ± 11.5 [65.2–67.0]	82.8 ± 10.9 [81.0-84.6]	65.8 ± 11.6 [64.1–67.5]
BMI (kg/m^2^)	26.3 ± 3.4 [25.8–26.8]	24.5 ± 4.3 [23.9–25.2]	26.2 ± 3.3 [25.7–26.7]	24.3 ± 4.1 [23.7–24.9]
FM (kg)	21.3 ± 6.9 [20.2–22.4]	23.5 ± 8.4 [22.2–24.8]	20.8 ± 6.4 [20.3–21.3]	23.4 ± 8.5 [22.2–24.6]
FFM (kg)	62.4 ± 7.2 [61.3–63.6]	42.6 ± 4.7 [41.9–43.3]	62.0 ± 6.9 [60.9–63.1]	42.4 ± 4.9 [41.7–43.1]
FMI (kg/m^2^)	6.7 ± 2.1 [6.4-7.0]	8.7 ± 3.2 [8.2–9.2]	6.6 ± 2.1 [6.3–6.9]	8.7 ± 3.2 [8.2–9.2]
FFMI (kg/m^2^)	19.6 ± 1.8 [19.3–19.9]	15.8 ± 1.6 [15.6–16.0]	19.6 ± 1.8 [19.3–19.9]	15.7 ± 1.5 [15.5–15.9]
PhA (°)	5.5 ± 0.6 [5.4–5.6]	4.9 ± 0.5 [4.8-5.0]	5.5 ± 0.6 [5.4–5.6]	4.9 ± 0.5 [4.8-5.0]
Physical activity				
HA (min/week)	606 ± 464 [531–681]*	537 ± 407 [475–599]*	458 ± 302 [409–507]*	439 ± 282 [398–480]*
SA (min/week)	193 ± 190 [162–224]	213 ± 198 [183–243]	171 ± 156 [146–197]	191 ± 167 [167–215]
Physical fitness				
Strength (z-Score^a^)	89.0 ± 12.3 [87.0–91.0]*	65.9 ± 8.1 [64.7–67.1]*	84.9 ± 11.7 [83.0-86.8]*	62.6 ± 9.2 [61.3–64.0]*
Coordination (z-Score)	83.2 ± 8.9 [81.8–84.6]*	79.6 ± 8.9 [78.3–81.0]*	90.3 ± 11.0 [88.5–92.1]*	85.2 ± 11.4 [83.5–86.9]*
Flexibility (z-Score)	96.0 ± 9.6 [94.5–97.5]	104.8 ± 9.8 [103.3-106.3]	99.0 ± 9.9 [97.4-100.7]	106.3 ± 8.6 [105.1-107.6]
CRF (z-Score)	96.9 ± 10.8 [95.2–98.6]	85.2 ± 4.4 [84.5–85.9]	95.3 ± 13.3 [93.1–97.5]	84.6 ± 4.7 [83.9–85.3]

n, sample size; SD, standard deviation; 95% CI, 95% confidence interval; cm, centimeter; kg; kilogram; BMI, body mass index; m, meter; FM, fat mass; FFM, fat-free mass; FMI, fat mass index; FFMI, fat-free mass index; PhA, phase angle; °, degree; HA, habitual activity; min, minutes; SA, sport activity; CRF, cardiorespiratory fitness.

^a^z-standardized variable.

*Significant difference (*p*<.05) between 2021 and 2025 (same sex).

### Regression analyses

In males, age was negatively associated with FFMI and PhA but not FMI. Habitual and sport activity were not associated with any outcomes. Strength was positively associated with FMI, FFMI, and PhA, whereas coordination was negatively associated with FMI and FFMI. CRF was positively associated with PhA. After adjusting for age, associations with FMI and FFMI persisted, but PhA was no longer associated with strength and CRF. In fully adjusted models, strength remained positively and coordination negatively associated with FMI and FFMI, while age was the sole negative predictor of PhA.

In females, age was inversely associated with FFMI and PhA but not FMI. Sport activity was positively associated with PhA in the age-adjusted model. Strength was positively associated with PhA, and coordination was negatively associated with FMI. After adjusting for age, strength was also negatively associated with FMI. In the fully adjusted model, strength remained negatively associated with FMI, age remained negatively associated with FFMI, and sport activity was positively associated with FFMI. PhA was inversely related to age and positively associated with strength and sport activity.

Please refer to Table 2 for results from regression models.

**Table 2:** The adjusted total coefficient of determination (R^2^ adj.) and the unstandardized regression coefficients (β) of age, PA, and PF in relation to FMI, FFMI, and PhA among males and females.

	Males						Females					
	FMI [kg/m^2^]		FFMI [kg/m^2^]		PhA [°]		FMI [kg/m^2^]		FFMI [kg/m^2^]		PhA [°]	
	R^2^	ß [95% CI]	R^2^	ß [95% CI]	R^2^	ß [95% CI]	R^2^	ß [95% CI]	R^**2**^	ß [95% CI]	R^2^	ß [95% CI]
Model 0	< 0.01		0.04		0.45		< 0.01		0.07		0.21	
Age (year)		0.12 [-0.04, 0.28]		-0.21* [-0.37, -0.05]		-0.67* [-0.79, -0.55]		0.12 [-0.03, 0.27]		-0.27* [-0.42, -0.12]		-0.46* [-0.59, -0.32]
Model 1	< 0.01		< 0.01		0.01		< 0.01		< 0.01		< 0.01	
HA (min/week)		0.02 [-0.16, 0.20]		0.02 [-0.16, 0.19]		-0.15 [-0.32, 0.03]		-0.11 [-0.26, 0.05]		0.02 [-0.14, 0.17]		0.00 [-0.15, 0.16]
SA (min/week)		-0.05 [-0.23, 0.12]		0.05 [-0.13, 0.23]		-0.03 [-0.21, 0.14]		-0.07 [-0.22, 0.08]		0.08 [-0.07, 0.23]		0.08 [-0.07, 0.24]
Model 1.1	< 0.01		0.04		0.45		0.02		0.08		0.23	
Age (year)		0.14 [-0.03, 0.31]		-0.25* [-0.41, -0.08]		-0.68* [-0.81, -0.56]		0.15 [-0.01, 0.30]		-0.30* [-0.45, -0.15]		-0.50* [-0.64, -0.37]
HA (min/week)		0.00 [-0.18, 0.18]		0.05 [-0.12, 0.22]		-0.05 [-0.19, 0.08]		-0.10 [-0.25, 0.05]		0.00 [-0.15, 0.15]		-0.02 [-0.15, 0.12]
SA (min/week)		-0.08 [-0.26, 0.10]		0.10 [-0.08, 0.27]		0.10 [-0.04, 0.23]		-0.10 [-0.26, 0.05]		0.15 [-0.01, 0.30]		0.20* [0.06, 0.34]
Model 2	0.11		0.08		0.29		0.12		0.03		0.16	
Strength (z-Score^a^)		0.26* [0.07, 0.46]		0.31* [0.11, 0.50]		0.40* [0.23, 0.57]		-0.17 [-0.36, 0.02]		0.13 [-0.08, 0.33]		0.31* [0.12, 0.50]
Coordination (z-Score)		-0.29* [-0.46, -0.11]		-0.20* [-0.38, -0.02]		0.05 [-0.11, 0.21]		-0.20* [-0.39, -0.01]		-0.08 [-0.28, 0.13]		0.04 [-0.15, 0.23]
Flexibility (z-Score)		-0.15 [-0.31, 0.03]		0.09 [-0.08, 0.26]		0.02 [-0.13, 0.17]		-0.10 [-0.25, 0.06]		0.08 [-0.09, 0.24]		0.00 [-0.16, 0.15]
CRF (z-Score)		-0.14 [-0.36, 0.01]		0.00 [-0.18, 0.18]		0.20* [0.04, 0.36]		0.01 [-0.17, 0.18]		0.15 [-0.03, 0.33]		0.15 [-0.02, 0.32]
Model 2.1	0.11		0.08		0.45		0.13		0.06		0.22	
Age (year)		0.15 [-0.07, 0.37]		-0.11 [-0.33, 0.11]		-0.57* [-0.74, -0.39]		-0.16 [-0.34, 0.03]		-0.23* [-0.42, -0.03]		-0.33* [-0.51, -0.16]
Strength (z-Score)		0.35* [0.12, 0.58]		0.25* [0.01, 0.48]		0.08 [-0.10, 0.26]		-0.21* [-0.41, -0.01]		0.07 [-0.14, 0.27]		0.22* [0.03, 0.41]
Coordination (z-Score)		-0.29* [-0.46, -0.11]		-0.20* [-0.38, -0.02]		0.05 [-0.09, 0.19]		-0.22* [-0.41, -0.02]		-0.11 [-0.31, 0.10]		0.00 [-0.19, 0.18]
Flexibility (z-Score)		-0.17 [-0.32, 0.02]		0.09 [-0.08, 0.26]		0.03 [-0.10, 0.16]		-0.11 [-0.26, 0.05]		0.06 [-0.11, 0.22]		-0.04 [-0.18, 0.11]
CRF (z-Score)		-0.16 [-0.32, 0.05]		-0.03 [-0.22, 0.16]		0.05 [-0.09, 0.20]		-0.05 [-0.23, 0.14]		0.08 [-0.11, 0.27]		0.04 [-0.13, 0.21]
Model 3	0.10		0.10		0.45		0.13		0.07		0.24	
Age (year)		0.15 [-0.07, 0.37]		-0.14 [-0.36, 0.08]		-0.58* [-0.75, -0.40]		-0.16 [-0.35, 0.04]		-0.29* [-0.49, -0.09]		-0.41* [-0.59, -0.22]
HA (min/week)		0.03 [-0.14, 0.20]		0.08 [-0.09, 0.25]		-0.05 [-0.18, 0.08]		-0.08 [-0.22, 0.07]		0.02 [-0.13, 0.17]		-0.02 [-0.15, 0.12]
SA (min/week)		-0.02 [-0.20, 0.15]		0.14 [-0.03, 0.32]		0.11 [-0.03, 0.25]		0.01 [-0.15, 0.16]		0.16* [0.00, 0.32]		0.18* [0.04, 0.32]
Strength (z-Score)		0.35* [0.11, 0.59]		0.31* [0.07, 0.55]		0.11 [-0.08, 0.29]		-0.22* [-0.42, -0.02]		0.05 [-0.16, 0.26]		0.20* [0.01, 0.39]
Coordination (z-Score)		-0.29* [-0.47, -0.11]		-0.21* [-0.39, -0.03]		0.06 [-0.08, 0.20]		-0.20 [-0.40, 0.01]		-0.15 [-0.35, 0.06]		-0.04 [-0.23, 0.15]
Flexibility (z-Score)		-0.15 [-0.32, 0.02]		0.08 [-0.09, 0.25]		0.03 [-0.11, 0.16]		-0.11 [-0.27, 0.05]		0.04 [-0.13, 0.20]		-0.06 [-0.21, 0.09]
CRF (z-Score)		-0.13 [-0.33, 0.06]		-0.06 [-0.26, 0.13]		0.03 [-0.12, 0.18]		-0.05 [-0.23, 0.13]		0.09 [-0.10, 0.27]		0.05 [-0.12, 0.22]

FMI, fat mass index; kg, kilogram; m, meter; FFMI, fat-free mass index; PhA, phase angle; °, degree; R^2^, adjusted coefficient of determination; ß, beta coefficient; 95% CI, 95% confidence interval; HA, habitual activity; min, minutes; SA, sport activity; CRF, cardiorespiratory fitness.

^a^z-standardized variable.

*ß is significant at the 0.05 level.

## Discussion

Our study shows that BC derived from BIA in a representative sample of middle-aged and older adults from a rural community in Germany remained stable from 2021^5^ to 2025. Key parameters such as body mass index (BMI), FMI, FFMI, and PhA did not differ significantly across waves. Regression analyses confirmed a largely unchanged pattern of associations. Age remained a central predictor of BC, with the strongest link to PhA in males, and PF continued to show stronger associations with BC than self-reported PA.

Mean BMI and FMI values remained stable from 2021 to 2025 and were consistent with national data from Germany^14^.

During the COVID-19 pandemic, nationally representative data from Germany^15^ indicated that active travel, assessed via walking and cycling, remained largely stable, particularly among older adults, suggesting that pandemic-related restrictions did not result in a substantial decline in everyday activity. In contrast, the post-pandemic increase in habitual activity observed in the present study may reflect a reorganization of daily routines, increased health awareness, and sustained engagement in habitual activity following the lifting of pandemic-related restrictions.

The divergent trajectories of muscular strength and coordination may be partially explained by shifts in resistance training modalities during and after the pandemic. While most individuals who engaged in resistance training prior to COVID-19 continued training during lockdowns, this period was characterized by home-based, bodyweight-oriented training with higher repetition ranges^16^. A subsequent return to machine-based training and higher external loads in local gyms may have promoted strength gains while reducing coordinative requirements, potentially contributing to the observed pattern.

The key patterns reported in 2021 were fully replicated in the 2025 cohort. First, across both waves, the strongest association was consistently observed between age and PhA, particularly among males. This underscores PhA as a sensitive marker of age-related biological decline^17^. Second, PA repeatedly showed only minimal explanatory value for BC. Habitual activity was not associated with FMI, FFMI, and PhA in both sexes and across both assessment waves. Sport activity also remained unassociated with BC markers among males, consistent with 2021 results. Among females, sport activity was the only PA dimension showing statistically significant associations with BC, confirming the previously observed sex-specificity of PA–BC associations^5^. Third, PF continued to explain substantially more of BC variance than PA. Strength emerged as the strongest and most consistent PF correlate across outcomes and sexes, showing the highest associations across both waves with PhA. This outcome demonstrates that muscular strength and PhA may reflect shared underlying properties of muscle tissue^17,18^.

Although the overall structure of associations remained stable, minor deviations were observed in 2025, such as changes in the associations of age, sport activity, CRF, and coordination with BC. These likely reflect post-pandemic behavioral shifts or minor cohort-specific factors without affecting the core findings.

Several limitations should be considered when interpreting the present findings. Our sample was drawn from a single rural community, which may limit the external validity of the results. However, the mean BMI observed in our sample was comparable to representative German data reported by the Robert Koch Institute. Furthermore, due to the cross-sectional design, no causal inferences can be drawn. PA was assessed using self-reports, which are associated with several problems such as recall bias^19^ and over- or underestimation of activity levels^20^. However, as we used the exact same methodological approach, we do not expect these errors to substantially affect the comparison between waves in the present study. Lastly, both study waves were conducted during the late spring and early summer months, which may have resulted in higher reported levels of PA due to seasonal effects when compared with studies conducted at other times of the year.

## Conclusion

Our findings confirm previously reported associations between BC, PA, and PF in middle-aged and older adults. Muscular strength and advanced age remain the variables most strongly associated with BC. Importantly, these associations were preserved across different societal contexts, underscoring their generalizability and relevance.

## Data Availability

The datasets used and/or analysed during the current study are available from the corresponding author on reasonable request.
